# A Drag Model-LIDAR-IMU Fault-Tolerance Fusion Method for Quadrotors

**DOI:** 10.3390/s19194337

**Published:** 2019-10-08

**Authors:** Pin Lyu, Bingqing Wang, Jizhou Lai, Shichao Liu, Zhimin Li

**Affiliations:** Navigation Research Center, Nanjing University of Aeronautics and Astronautics, Nanjing 210016, China; lvpin@nuaa.edu.cn (P.L.); wangbingqing@nuaa.edu.cn (B.W.); liushichao@nuaa.edu.cn (S.L.); lizhimin@nuaa.edu.cn (Z.L.)

**Keywords:** model-aided navigation, fault-tolerant estimation, improved drag model, quadrotor

## Abstract

In this paper, a drag model-aided fault-tolerant state estimation method is presented for quadrotors. Firstly, the drag model accuracy was improved by modeling an angular rate related item and an angular acceleration related item, which are related with flight maneuver. Then the drag model, light detection and ranging (LIDAR), and inertial measurement unit (IMU) were fused based on the Federal Kalman filter frame. In the filter, the LIDAR estimation fault was detected and isolated, and the disturbance to the drag model was estimated and compensated. Some experiments were carried out, showing that the velocity and position estimation were improved compared with the traditional LIDAR/IMU fusion scheme.

## 1. Introduction

In recent years, quadrotors have been widely used in many fields, such as agriculture, industry, and ecology [1]. Navigation systems calculate attitude, velocity, and position, which are important for stable control. For quadrotors, inertial navigation systems (INS) and global positioning system (GPS) integration systems are mostly used. However, GPS cannot be used in an indoor environment because of signal interference. In a GPS denied environment, vision [2] or light detection and ranging (LIDAR) [3] based navigation methods are usually adopted.

Vision navigation is based on computer vision algorithms. The environment is captured by the camera, then the relative motion between the camera and the environment is estimated. However, the estimation accuracy is usually affected by light interference [4]. In some dark and enclosed environments, such as caves and tunnels [5], a LIDAR-based navigation method is usually adopted. LIDAR is an active sensor and can measure ranges between itself and the environment. The motion state is estimated by using the simultaneous localization and mapping (SLAM) algorithm [6]. 

LIDAR can be divided into 2 dimensional (2D) LIDAR [7] and 3 dimensional (3D) LIDAR [8]. The 2D type can measure a two-dimensional plane, and the 3D type can measure three-dimensional space. Due to size and weight limitations, 2D LIDAR is usually adopted by quadrotors. Because quadrotors fly in a 3D environment, the classical 2D LIDAR SLAM may lead to estimation error [9]. It is usually assumed that the environment is formed by collections of vertical walls [10]. In some complex or sparse-feature environments, the state estimation accuracy will decrease. As a result, the robustness of the SLAM algorithm in these environments is now a challenging issue [11]. 

Dynamic model-aided navigation is a novel and developed method. The dynamic model of plants, which describes the relationship between the plant motion, control input, and surrounding environment, is fused with navigation sensors, improving the navigation accuracy and reliability. The dynamic model-aided navigation method has been used for aircraft [12], vehicles [13], and underwater robots [14]. The fusion schemes for different plants are different because they should be designed based on the model characteristics. 

As for the fixed wing aircraft, the whole dynamic model (including the thrust, drag, and moments) is considered [15]. The dynamic model is fused with the INS. Experiment results show that the scheme is useful for the low-cost micro-electromechanical systems (MEMS) INS and can improve the navigation accuracy [16]. As the state estimation using the dynamic model is a dead reckoning process, the estimation accuracy is affected by the model parameter uncertainty [17]. 

The quadrotor is an underactuated system. The horizontal motion is coupled with the attitude motion, which is usually estimated by fusing gyros and accelerometers [18], and the drag is proportional to the velocity [19]. In recent years, drag model-aided navigation has been widely studied. [20] proposed a drag model/INS fusion scheme. The velocity and attitude estimation are improved compared with the pure INS scheme. [21] showed that the velocity estimation accuracy can be kept the same while the frequency of position correction decreases. A unified model technique was used by [22]. The state prediction of the dynamic model and INS are fused, and the procedure of the traditional Kalman filter is simplified. A dynamic model/INS/GPS fusion scheme was proposed by [23]. When GPS is available, the INS/GPS fusion results are used to identify the model parameters. When GPS is denied, the dynamic model is fused with INS. The dynamic model/INS/vision sensor fusion scheme is adopted by the AR. Drone [24]. The accelerometer’s bias can be estimated online. In [25], the dynamic model was used to estimate the scale factor of monocular vision. In [26], a dynamic model/optical flow/inertial sensor fault-tolerant fusion method was proposed. The faults of the above sensors can be detected. The navigation accuracy can be retained in the case of failure of one sensor. 

**Figure 1:**
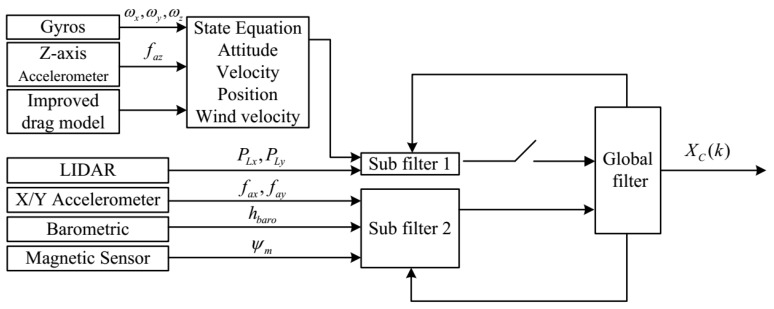
The architecture of the proposed fault-tolerant filter.

In this paper, a drag model-LIDAR-IMU fusion scheme (Figure 1) is proposed for quadrotors in an indoor environment. The contributions of the paper can be summarized as:

(1) An improved drag model of quadrotors is proposed. The traditional drag model only contains the velocity related item. Its accuracy is affected by maneuver flight [27]. In this paper, some attitude motion related items are considered to improve the model accuracy.

(2) A fault-tolerant state estimation is realized for quadrotors. The failures of LIDAR and drag model are both considered. If the LIDAR SLAM accuracy decreases due to environmental disturbance, it is detected and isolated from the global filter. The drag model accuracy may be affected by the wind. In the filter, the wind velocity is treated as a state and can be estimated online. Therefore, the accuracy decrease brought by the wind is suppressed.

## 2. Improved Drag Model of Quadrotor

**Figure 2:**
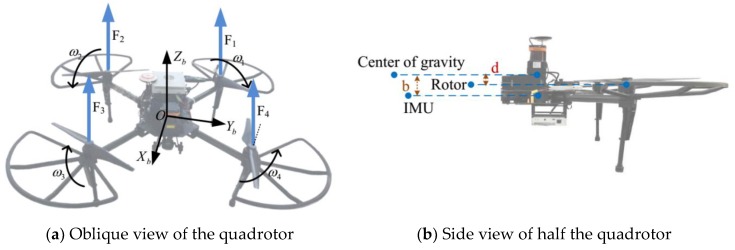
Quadrotor structure diagram.

In this paper, the body coordinate system (b-frame) is defined as the front-right-down frame (Figure 2), and the navigation coordinate system (n-frame) is defined as the local north-east-down frame. In the dynamic model-aided navigation method, the model accuracy affects the navigation performance. Therefore, the drag model will be studied in this section.

### 2.1. Drag Modeling of Quadrotor

The traditional drag model can be expressed as [18]

(1) $$\left\{ \begin{array}{l} D_{x}=k_{x}(V_{nbx}^{b}-V_{wx}^{b})\\ D_{y}=k_{y}(V_{nby}^{b}-V_{wy}^{b}) \end{array}\right.$$

where $D_{x}$ and $D_{y}$ represent the x-axis and y-axis drag force resolved in the b-frame. $V_{nbx}^{b}$ and $V_{nby}^{b}$ are the components of $V_{nb}^{b}$, which is the linear velocity of the b-frame with respect to the n-frame resolved in the b-frame. $V_{wx}^{b}$ and $V_{wy}^{b}$ are the components of $V_{w}^{b}$, which is the wind velocity resolved in the b-frame. $k_{x}$ and $k_{y}$ are the drag coefficients. 

Equation (1) means that the drag is proportional to the airspeed. If the wind is ignored, Equation (1) is transformed to

(2) $$\left\{ \begin{array}{l} D_{x}=k_{x}V_{nbx}^{b}\\ D_{y}=k_{y}V_{nby}^{b} \end{array}\right.,\;$$

Because the drag force can be estimated by the accelerometer, it can be derived as

(3) $$\left\{ \begin{array}{l} f_{nbx}^{b}m=k_{x}V_{nbx}^{b}\\ f_{nby}^{b}m=k_{y}V_{nby}^{b} \end{array}\right.,\;$$

where $f_{nbx}^{b}$ and $f_{nby}^{b}$ are the x-axis and y-axis accelerations resolved in the b-frame, which can be obtained from the outputs of the accelerometers. It can be seen that $V_{nbx}^{b}$ and $V_{nby}^{b}$ are proportional to $f_{nbx}^{b}$ and $f_{nby}^{b}$. 

In the traditional INS algorithm, velocity is calculated by integrating the accelerometers’ outputs. Using the drag model, the velocity can be directly estimated from the accelerometers. Therefore, the velocity error can be bounded.

In this paper, two additional factors are taken into account. Firstly, the traditional drag force model is derived based on the characteristics of one blade, and the quadrotor is treated as a mass point. However, there exists distances among the rotors, accelerometers, and center of gravity (CoG), shown in Figure 2. d represents the distance between the rotor and the CoG, and b represents the distance between the accelerometers and the CoG. When the quadrotor pitches or rolls, a tangential acceleration between the accelerometer and the center of gravity is introduced. Therefore, Equation (3) transforms to

(4) $$\left\{ \begin{array}{l} \left(f_{nbx}^{b}-{\dot{\omega }}_{nby}^{b}/b-f_{x0}\right)m=k_{x}V_{nbx}^{b}\\ \left(f_{nby}^{b}-{\dot{\omega }}_{nbx}^{b}/b-f_{y0}\right)m=k_{y}V_{nby}^{b} \end{array}\right.,$$

where $\omega_{nb}^{b}=\left[\begin{array}{ccc} \omega_{nbx}^{b} & \omega_{nby}^{b} & \omega_{nbz}^{b} \end{array}\right]$ is the angular rate of the b-frame with respect to the n-frame resolved in the b-frame, which can be estimated from gyros’ outputs. $f_{x0}$ and $f_{y0}$ are the biases of the x- and y-axis accelerometers.

Secondly, in the traditional drag model, the blade flapping effect is ignored, which is an important aspect of quadrotor dynamics [28]. The flapping effect causes the blade rotation plane to tilt, and the blade velocity can be expressed as [29]

(5) $$V_{ri}^{}=V_{nb}^{b}+\omega_{nb}^{b}\times L_{i},$$

where $V_{ri}^{}$ is the ith rotor’s velocity, and $L_{i}$ is the displacement between the rotor and the CoG. Therefore, the drag force of the *i*th rotor can be expressed as

(6) $$D_{ri}^{}=kV_{ri}^{}=k\left(V_{nb}^{b}+\omega_{nb}^{b}\times L_{i}\right),$$

Then the drag force of the whole quadrotor can be derived as

(7) $$\begin{array}{ll} D & =\sum_{i=1}^{4}D_{ri}^{}\\ & =\sum_{i=1}^{4}kV_{ri}^{}\\ & =k\left(4V_{nb}^{b}+\sum_{i=1}^{4}\omega_{nb}^{b}\times L_{i}\right)\\ & =4k\left(V_{nb}^{b}+{\left[\begin{array}{ccc} d\omega_{nby}^{b} & -d\omega_{nbx}^{b} & 0 \end{array}\right]}^{T}\right) \end{array}$$

Combining Equations (4) and (7), the improved drag model can be expressed as

(8) $$\left\{ \begin{array}{l} \left(f_{nbx}^{b}-{\dot{\omega }}_{nby}^{b}/b\right)m={k^{\prime }}_{x}\left(V_{nbx}^{b}+d\omega_{nby}^{b}\right)\\ \left(f_{nby}^{b}-{\dot{\omega }}_{nbx}^{b}/b\right)m={k^{\prime }}_{y}\left(V_{nby}^{b}-d\omega_{nbx}^{b}\right) \end{array}\right.,$$

Then the velocity can be derived as

(9) $$\left\{ \begin{array}{l} V_{nbx}^{b}=k_{x0}^{}+k_{x1}^{}f_{nbx}^{b}+k_{x2}^{}{\dot{\omega }}_{nby}^{b}+k_{x3}^{}\omega_{nby}^{b}\\ V_{nby}^{b}=k_{y0}^{}+k_{y1}^{}f_{nby}^{b}+k_{y2}^{}{\dot{\omega }}_{nbx}^{b}+k_{y3}^{}\omega_{nbx}^{b} \end{array}\right.,$$

where $k_{x0}^{}$, $k_{x1}^{}$, $k_{x2}^{}$, $k_{x3}^{}$, $k_{y0}^{}$, $k_{y1}^{}$, $k_{y2}^{}$, and $k_{y3}^{}$ are constant coefficients. 

It can be seen that there are four items in the improved drag model: the accelerometer bias, the velocity related item, the angular rate related item, and the angular acceleration related item. 

### 2.2. Test of Drag Model Accuracy

**Figure 3:**
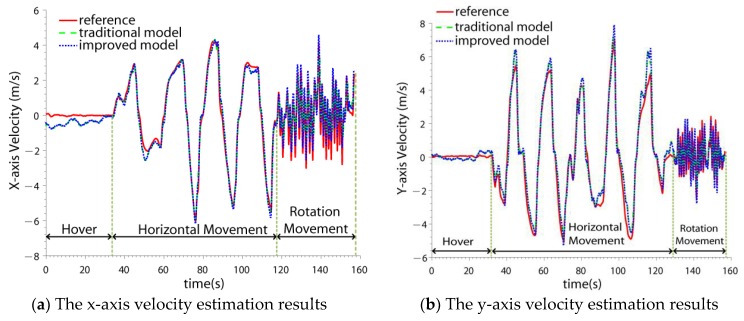
The comparison between the velocities estimated by different drag models.

Some experiments were done to verify the superiority of the improved model. The quadrotor was controlled to do different maneuvers, including hover, horizontal flight, and attitude rotation. Five experiments were conducted in a no wind environment. The least square method was used to identify the model parameters. The velocities estimated by the traditional model and the improved model were compared. The velocity obtained by GPS was treated as a reference. In the improved model, the angular acceleration was obtained by differentiating the gyros’ signals. Because the difference operation introduces noise to the signal, it was smoothed before being used. The accelerometers’ outputs were also used as inputs of the models. The experimental results are shown in Figure 3 and Table 1. Figure 3 is the velocity estimation result of one experiment, and Table 1 is the statistical results of the five experiments.

From the experiments, it can be seen that during the hover or horizontal flight, the accuracy of the two models are almost the same. When the quadrotor does an attitude motion, the accuracy of the improved model is better than the traditional model by 1.38 times in x-axis and 1.56 times in y-axis.

## 3. Drag Model-LIDAR-IMU Fusion Scheme

In this section, the fault-tolerant fusion scheme is studied. The drag model, IMU, and LIDAR were fused through a Federal Kalman filter (FKF). The filter can deal with the disturbances to the LIDAR and drag model.

### 3.1. Quadrotor Dynamic Equation

According to the drag model (considering the wind effect) and the INS algorithm, the velocity differential equation can be expressed as

(10) $$\left[\begin{array}{c} {\dot{V}}_{nbx}^{b}\\ {\dot{V}}_{nby}^{b}\\ {\dot{V}}_{nbz}^{b} \end{array}\right]=\left[\begin{array}{c} V_{nbx}^{b}\\ V_{nby}^{b}\\ V_{nbz}^{b} \end{array}\right]\times \left[\begin{array}{c} \omega_{nbx}^{b}\\ \omega_{nby}^{b}\\ \omega_{nbz}^{b} \end{array}\right]+\left[\begin{array}{c} (V_{nbx}^{b}-V_{wx}^{b}-k_{x0}-k_{x2}{\dot{\omega }}_{nby}^{b}-k_{x3}\omega_{nby}^{b})/k_{x1}\\ (V_{nby}^{b}-V_{wy}^{b}-k_{y0}-k_{y2}{\dot{\omega }}_{nbx}^{b}-k_{y3}\omega_{nbx}^{b})/k_{y1}\\ f_{nbz}^{b} \end{array}\right]+C_{n}^{b}\left[\begin{array}{c} 0\\ 0\\ g \end{array}\right],$$

where $f_{nbz}^{b}$ is the z-axis acceleration resolved in the b-frame, $C_{n}^{b}$ is the coordinate transformation matrix from the n-frame to the b-frame, and g is the gravitational acceleration.

The attitude is described through quaternion, and the attitude differential equation can be expressed as

(11) $$\left[\begin{array}{c} {\dot{q}}_{0}\\ {\dot{q}}_{1}\\ {\dot{q}}_{2}\\ {\dot{q}}_{3} \end{array}\right]=0.5\left[\begin{array}{cccc} 0 & -\omega_{nbx}^{b} & -\omega_{nby}^{b} & -\omega_{nbz}^{b}\\ \omega_{nbx}^{b} & 0 & \omega_{nbz}^{b} & -\omega_{nby}^{b}\\ \omega_{nby}^{b} & -\omega_{nbz}^{b} & 0 & \omega_{nbx}^{b}\\ \omega_{nbz}^{b} & \omega_{nby}^{b} & -\omega_{nbx}^{b} & 0 \end{array}\right]\left[\begin{array}{c} q_{0}\\ q_{1}\\ q_{2}\\ q_{3} \end{array}\right],$$

where $q_{0}$, $q_{1}$, $q_{2}$, $q_{3}$ stand for the quaternion.

The position differential equation is

(12) $$\left[\begin{array}{c} {\dot{P}}_{N}\\ {\dot{P}}_{E}\\ {\dot{P}}_{D} \end{array}\right]=C_{b}^{n}\left[\begin{array}{c} V_{nbx}^{b}\\ V_{nby}^{b}\\ V_{nbz}^{b} \end{array}\right],$$

The wind velocity is regarded as constant velocity expressed as

(13) $$\left[\begin{array}{c} {\dot{V}}_{wx}^{b}\\ {\dot{V}}_{wy}^{b} \end{array}\right]=\left[\begin{array}{c} 0\\ 0 \end{array}\right],$$

### 3.2. Fault-Tolerant Filter Design

The architecture of the filter is shown in Figure 1. It contains a main filter and two sub-filters, which are denoted as C1 and C2, respectively. The wind velocity affects the drag model accuracy, thus it is included in the state vector, which is chosen as

(14) $$x_{c}={\left[\begin{array}{cccccccccccc} q_{0} & q_{1} & q_{2} & q_{3} & V_{nbx}^{b} & V_{nby}^{b} & V_{nbz}^{b} & P_{N} & P_{E} & P_{D} & V_{wx}^{b} & V_{wy}^{b} \end{array}\right]}^{T},$$

The input vector is defined as

(15) $$u={\left[\begin{array}{ccccccc} \omega_{x} & \omega_{y} & \omega_{z} & f_{az} & {\dot{\omega }}_{x} & {\dot{\omega }}_{y} & g \end{array}\right]}^{T},$$

where $\omega_{x}$, $\omega_{y}$, and $\omega_{z}$ are the x, y, and z axis gyros’ outputs, $f_{az}$ is the z-axis accelerometer’s output, and g is the gravitational acceleration. 

The state equation can be derived from (10)–(13) and expressed as

(16) $$\dot{x}=f(x,u)+Gw,$$

where G is the noise transition matrix, and w is the state noise matrix. 

The measurements of the two sub-filters are chosen as

(17) $$\left\{ \begin{array}{l} z_{1}=\left[\begin{array}{cc} P_{Lx} & P_{Ly} \end{array}\right]\\ z_{2}=\left[\begin{array}{cccc} f_{ax} & f_{ay} & \psi_{m} & h_{baro} \end{array}\right] \end{array}\right.,$$

where $P_{Lx}$ and $P_{Ly}$ are the position estimated by LIDAR SLAM method, $f_{ax}$ and $f_{ay}$ are the outputs of the x- and y-axis accelerometers, $\psi_{m}$ is the yaw estimated by the magnetic sensor, and $h_{baro}$ is the height estimated by the barometer.

The measurement equations of the two sub-filters are expressed as

(18) $$\left\{ \begin{array}{l} z_{1}=h_{c1}(x_{c})+V_{C1}\\ z_{2}=h_{c2}(x_{c})+V_{C2} \end{array}\right.,$$

where $V_{C1}$ and $V_{C2}$ are the measurement noise matrices of C1 and C2. The updating progress of the FKF can be referred to in [30] and is not described here.

### 3.3. Fault Detection of LIDAR SLAM 

In this paper, the LIDAR SLAM fault is considered. The LIDAR SLAM accuracy is affected by the feature salience of the environment. In some sparse-feature environments, the accuracy is low. In that case, the LIDAR SLAM result should be cut off from the global filter. Although the wind introduces disturbance to the drag model, it is estimated in the filter, so the drag model fault is not considered. 

The chi-square test [31] is used for the fault detection of sub-filter C1. The statistics parameter is defined as

(19) $$\lambda_{C1}(k)=r_{C1}(k)P_{C1r}{\left(k\right)}^{-1}r_{C1}{\left(k\right)}^{T},$$

where $r_{C1}(k)$ is the residual of C1, and $P_{C1r}(k)$ is the covariance matrix of $r_{C1}(k)$. They are defined as

(20) $$r_{C1}(k)=z_{1}(k)-h_{c1}(x_{c}(k|k-1)),$$

(21) $$P_{C1r}(k)=H_{C1}(k)P_{C1}(k|k-1)H_{C1}{\left(k\right)}^{T}+R_{C1}(k),$$

where $x_{c}(k|k-1)$ is the prediction state, $P_{C1}(k|k-1)$ is the covariance matrix of $x_{c}(k|k-1)$, $H_{C1}(k)$ is Jacobian matrix and calculated by $H_{C1}(k)=\frac{\partial h_{c1}(x_{C}(k|k-1))}{\partial x_{C}(k|k-1)}$, and $R_{C1}(k)$ is the measuring noise variance matrix.

When there is no fault, $\lambda_{C1}(k)$ follows a chi-square distribution [30]. The fault detection function can be constructed as

(22) $$T_{D}(k)=\left\{ \begin{array}{ll} 1 & \lambda_{C1}(k)>\tau_{D}\;\\ 0 & \lambda_{C1}(k)<\tau_{D} \end{array}\right.,$$

where $\tau_{D}$ is the threshold. $T_{D}(k)=1$ means that the LIDAR SLAM error increases and should be isolated from the filter. 

### 3.4. Observability Analysis

The observability is an important indicator for a filter. It can reflect whether the states can be estimated. In this paper, the lie deviation method [32] is adopted for observability analysis. Two cases are considered: (1) no fault occurs; (2) the position supplied by LIDAR SLAM is faulty. 

When there is no fault, the rank of the observability matrix is 12. It means that all the states are observable. However, when the position measurement is not available the rank is 8. It means that 4 states are not observable. Using the null space analysis method [33], the unobservable states are $\left[\begin{array}{cccc} P_{N} & P_{E} & V_{wx}^{b} & V_{wy}^{b} \end{array}\right]$, which are the horizontal position and wind velocity. 

## 4. Experiments and Analysis

In this section, experiments designed and carried out to test the proposed method are described. The following cases were considered:

(1) The navigation result in the LIDAR SLAM failure case. The navigation performance of the proposed method needs to be tested.
(2) The test when the quadrotor does an attitude maneuver. The proposed drag model improves compared with the traditional model, so the navigation accuracy should be tested.
(3) The test under wind. The wind introduces interference to the model, so the navigation accuracy in windy environment should be considered.

### 4.1. Test Setup

The test platform was built based on a DJI M100 quadrotor. The quadrotor specification is shown in Table 2. The navigation system included an IMU, a magnetic sensor, a barometer, and a 2D LIDAR. The navigation result was outputted to the autopilot N1 and served as the control algorithm. The update rates of IMU, magnetic sensor, and barometer were 50 Hz, and the update rate of the LIDAR SLAM was 10 Hz.

**Figure 4:**
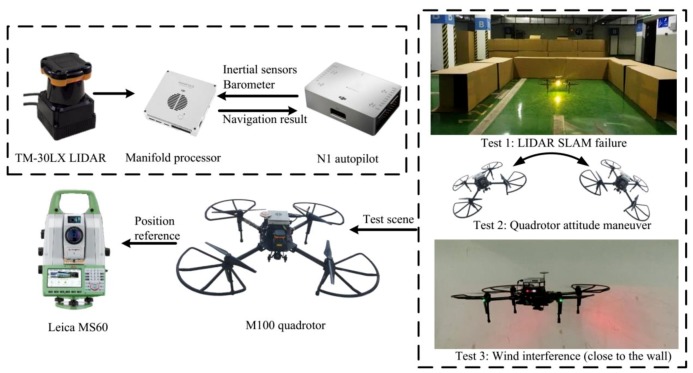
The test scheme.

The test scheme is shown in Figure 4. The experiments were carried out in an underground garage. A total station (Leica MS60) was used as the position reference. The position accuracy was better than 0.01 m. 

### 4.2. Test in LIDAR SLAM Failure Case 

**Figure 5:**
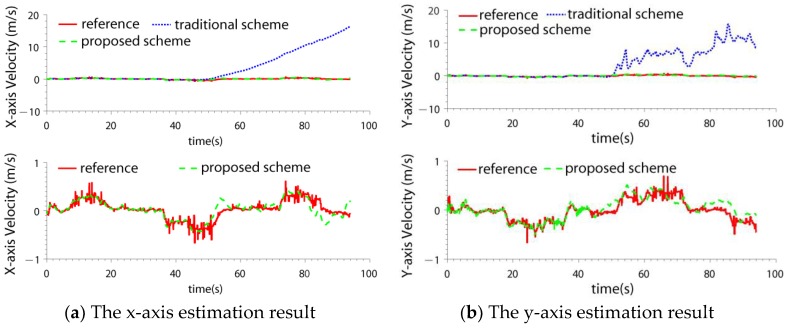
The velocity estimation result in light detection and ranging (LIDAR) simultaneous localization and mapping (SLAM) failure case.

**Figure 6:**
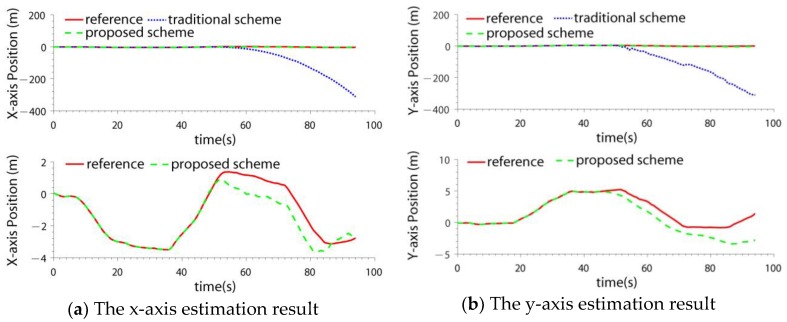
The position estimation result in LIDAR SLAM failure case.

**Figure 7:**
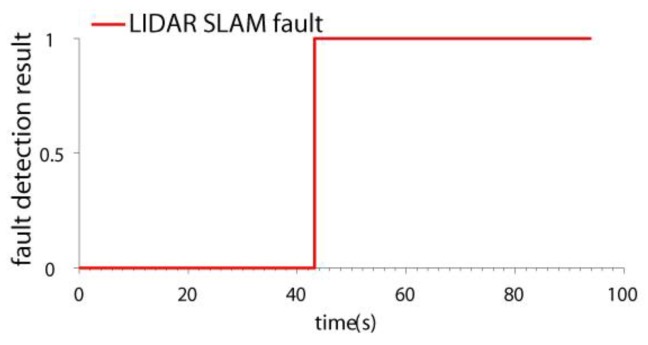
The fault detection results in LIDAR SLAM failure case.

In this paper, the ICP (Iterated Closest Points) LIDAR SLAM algorithm [34,35] was adopted. It is a classical SLAM method. Because the SLAM method for 2D LIDAR is based on the 2.5D assumption [9,10], when there exists a step change in the environment, position estimation error may be introduced. In the test, some carton boxes were placed to construct the environment changes (shown in Figure 4). When the quadrotor flew across the boxes, the LIDAR SLAM failed. The velocity and position estimation results of two schemes are compared: the IMU/LIDAR fusion scheme and the proposed drag model-LIDAR-IMU fusion scheme (shown in Figure 5 and Figure 6). The fault detection result is shown in Figure 7. The RMSE of velocity and position is shown in Table 3.

From the experimental results, it can be seen that:

(1) When the quadrotor flew over the boxes, the LIDAR SLAM algorithm failed due to a step environment change. The LIDAR SLAM failure can be detected and isolated by both the two schemes.
(2) When the LIDAR was isolated from the filter, the IMU/LIDAR fusion scheme degraded to the pure INS scheme. The navigation accuracy improved by introducing the drag model. The velocity error was bounded, and the positioning error also significantly decreased. The x-axis and y-axis velocity accuracies improved by 54.6 times and 51.0 times, respectively. The x-axis and y-axis position accuracies improved by 135.5 times and 78.1 times, respectively.

### 4.3. Quadrotor Attitude Maneuver Test

**Figure 8:**
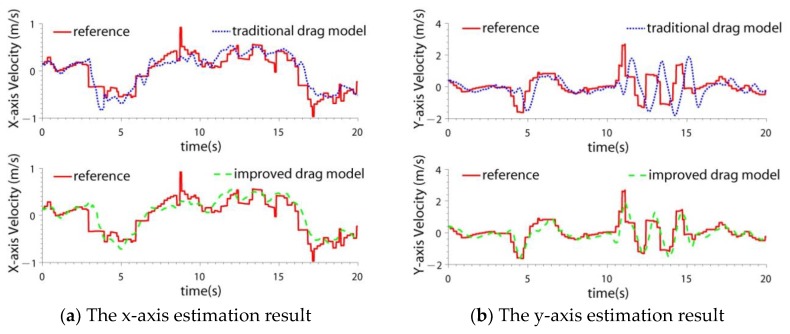
The velocity estimation result in quadrotor attitude maneuver case.

**Figure 9:**
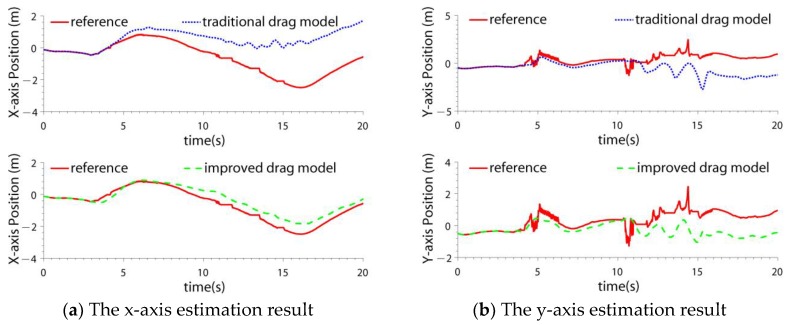
The position estimation result in quadrotor attitude maneuver case.

**Figure 10:**
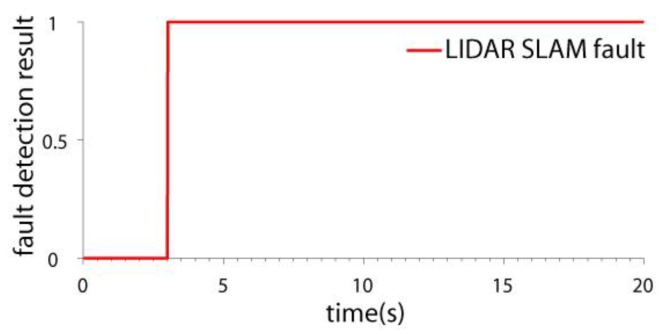
The fault detection results in quadrotor attitude maneuver case.

The quadrotor was controlled to do attitude maneuvers. The traditional drag model and the improved drag model were compared. The navigation results are shown in Figure 8, Figure 9, and Table 4. The fault detection result is shown in Figure 10.

From the experimental results, it can be seen that:

(1) When the quadrotor completed attitude maneuvers, the LIDAR SLAM accuracy decreased and failed. This was due to the mismatch of the LIDAR scanned points.
(2) In the test, the quadrotor completed an attitude maneuver in the y-axis, so the y-axis velocity accuracy improved by 2.3 times using the improved model, while the x-axis velocity accuracies of the two models were almost the same. The percentage increase of the x-axis position accuracy (3.9 times) was larger than the y-axis position (1.5 times), that is because the velocity errors of the y-axis velocity were offset after the integration.
(3) It was noticed that the accuracy improvement (2.3 times) was different from the test result of the y-axis velocity in Section 2.2, which was 1.56 times. That is because the flight maneuvers of the two tests were different, which affected the improvement degree.

### 4.4. Wind Interference Test 

**Figure 11:**
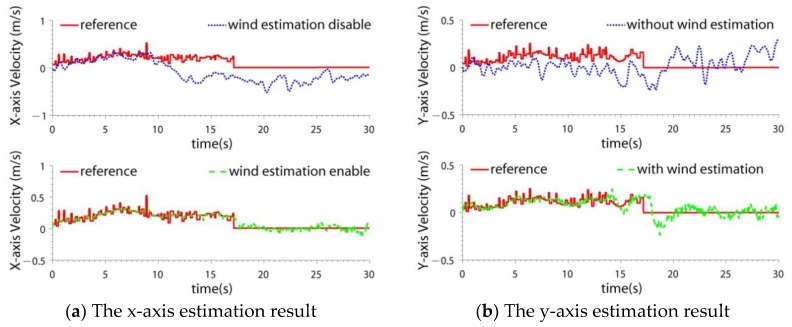
The velocity estimation result in the wind interference case.

**Figure 12:**
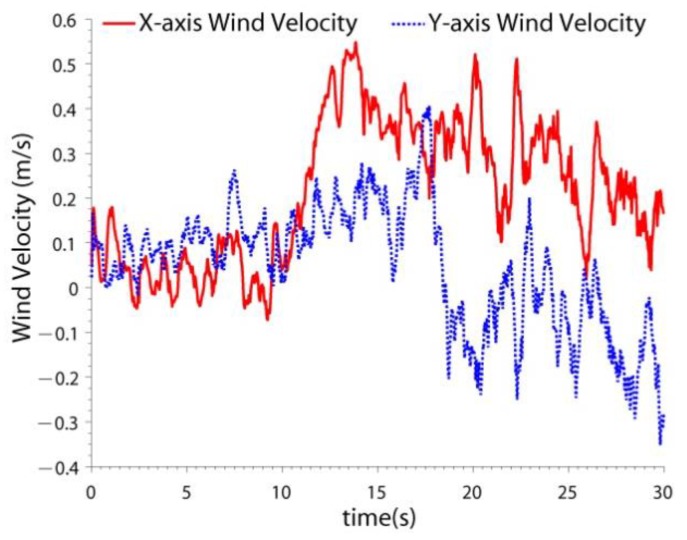
The wind estimation results.

Although the experiments were carried out indoors, wind interference may still be present. For example, when the quadrotor flew close to the wall (Figure 4), the wind force generated by the blades reacted on the quadrotor. Because wind does not affect the LIDAR SLAM, the accuracy of LIDAR SLAM was not tested. The navigation results of two schemes were compared: the drag model-LIDAR-IMU fusion filter with and without wind estimation. The velocity estimation results are shown in Figure 11 and the RMSE is shown in Table 5. The wind estimation results are shown in Figure 12.

From Figure 11 and Figure 12 and Table 5, it can be seen that:

(1) When the quadrotor flew near the wall, the velocity estimation accuracy decreased. That is because the wind introduces interference to the drag model. If the wind velocity is included in the state, the wind can be estimated, and the interference can be partly compensated. The x-axis velocity accuracy improved by 5.4 times and the y-axis velocity accuracy improved by 2.4 times.
(2) It can be seen that when the quadrotor was away from the wall, the estimated wind velocity was small (0 s~10 s). When the quadrotor flew close to the wall, the wind became greater. Because wind is generated by the reaction of the rotating blades, the estimated wind is not constant.

## 5. Conclusions

A drag model-LIDAR-IMU fault-tolerant fusion method was proposed. An angular rate related item and an angular acceleration related item were added to the traditional drag model. The model accuracy during the maneuver flight improved. An FKF based fusion scheme was designed. The LIDAR SLAM estimation fault was detected and isolated from the filter, avoiding the disturbance to the navigation result. Compared with the traditional method, the velocity and position accuracy is improved by introducing the drag model. The wind velocity is included in the states and can be estimated online, and the filter is robust to wind interference.

## Figures and Tables

**Table 1:** The velocity RMSE (root mean square error) comparison of different drag models.

State	X-axis Velocity RMSE (m/s)		Y-axis Velocity RMSE (m/s)	
	Traditional Drag Model	Improved Drag Model	Traditional Drag Model	Improved Drag Model
**Hover**	0.455	0.443	0.190	0.189
**Horizontal movement**	0.288	0.267	0.573	0.554
**Rotation movement**	0.908	0.655	0.837	0.534

**Table 2:** Unmanned aerial vehicle (UAV) technical features.

Technical Features	Description
Airframe	DJI-M100 Arm length 0.65 m
Autopilot	DJI N1
2D LIDAR	Hokuyou TM-30LX, Scanning range 30 m
Navigation processor	DJI Manifold

**Table 3:** The RMSE (root mean square error) comparison of different schemes.

State	X-axis Velocity RMSE (m/s)	Y-axis Velocity RMSE (m/s)	X-axis Position RMSE (m)	Y-axis Position RMSE (m)
**Traditional Scheme**	8.020	7.503	121.832	147.137
**Proposed Scheme**	0.147	0.147	0.899	1.885

**Table 4:** The RMSE (root mean square error) comparison of different drag model.

State	X-axis Velocity RMSE (m/s)	Y-axis Velocity RMSE (m/s)	X-axis Position RMSE (m)	Y-axis Position RMSE (m)
**Traditional Drag Model**	0.192	0.975	1.631	1.388
**Improved Drag Model**	0.188	0.422	0.414	0.952

**Table 5:** The RMSE (root mean square error) comparison of different wind estimation.

State	X-axis Velocity RMSE (m/s)	Y-axis Velocity RMSE (m/s)
**Wind Estimation Disable**	0.264	0.141
**Wind Estimation Enable**	0.049	0.058
